# Supplementary material for: Liver ACOX1 regulates levels of circulating lipids that promote metabolic health through adipose remodeling
Source: Nat Commun. 2024 May 17;15:4214. doi: 10.1038/s41467-024-48471-2 (PMC11101658; doi:10.1038/s41467-024-48471-2)
Supplement: Supplementary file 1 — Supplementary Information [file 41467_2024_48471_MOESM1_ESM.pdf]

Supplementary Information

Liver ACOX1 Regulates Levels of Circulating Lipids that Promote Metabolic Health through Adipose Remodeling

Dongliang Lu, Anyuan He, Min Tan, Marguerite Mrad, Amal El Daibani, Donghua Hu, Xuejing Liu, Brian Kleiboeker, Tao Che, Fong-Fu Hsu, Monika Bambouskova, Clay F. Semenkovich, and Irfan J. Lodhi

Supplementary information includes six Supplementary Figures and one Supplementary Table.

Supplementary Fig. 1. Characterization of glucose homeostasis and immune cells in Acox1-LKO and control mice.

Supplementary Fig. 2. Metabolic phenotyping and adipose tissue gene expression in Acox1-LKO and control mice.

Supplementary Fig. 3. Liver and serum lipidomic analysis in Acox1-LKO and control mice.

Supplementary Fig. 4. Effect of ω-3 VLCFAs on browning of adipocytes derived from human WAT.

Supplementary Fig. 5. Effect of ω-3 VLCFAs on PPARγ activation and GPR120 binding.

Supplementary Fig. 6. Pharmacological inhibition of ACOX1 promotes adipose tissue browning and protects mice against diet-induced obesity and insulin resistance.

Supplementary Table 1. Oligonucleotide sequences

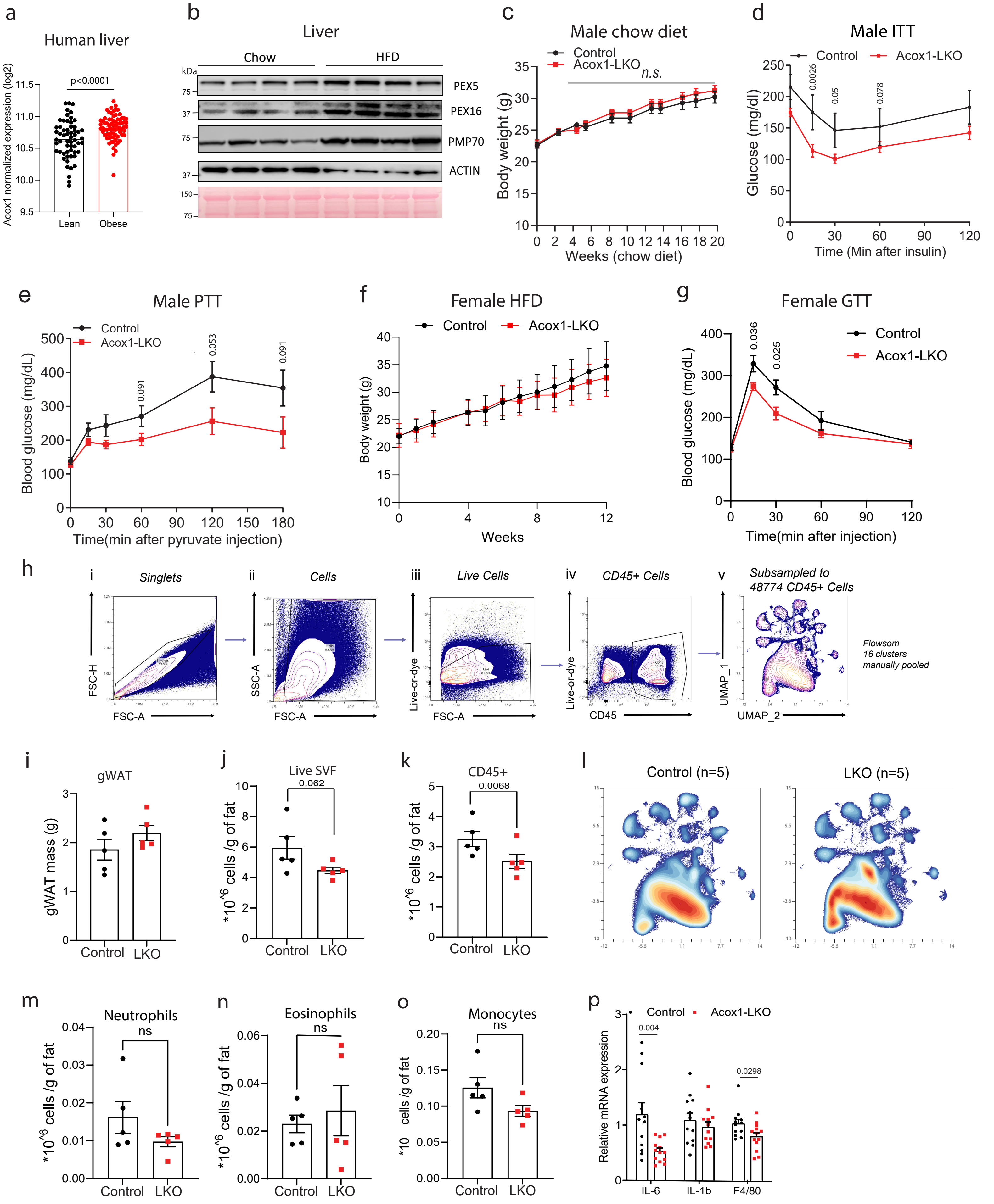
a
Human liver
p<0.0001
Acox1 normalized expression (log2)
Lean
Obese
b
Liver
Chow
HFD
kDa
PEX5
PEX16
PMP70
ACTIN
c
Male chow diet
Control
Acox1-LKO
n.s.
Body weight (g)
Weeks (chow diet)
d
Male ITT
Control
Acox1-LKO
0.0026
0.05
0.078
Glucose (mg/dl)
Time (Min after insulin)
e
Male PTT
Control
Acox1-LKO
0.091
0.053
0.091
Blood glucose (mg/dL)
Time(min after pyruvate injection)
f
Female HFD
Control
Acox1-LKO
Body weight (g)
Weeks
g
Female GTT
Control
Acox1-LKO
0.036
0.025
Blood glucose (mg/dL)
Time(min after injection)
h
i
Singlets
ii
Cells
iii
Live Cells
iv
CD45+ Cells
v
Subsampled to 48774 CD45+ Cells
FSC-H
FSC-A
SSC-A
Live-or-dye
CD45
UMAP_1
UMAP_2
Flowsom 16 clusters manually pooled
i
gWAT
gWAT mass (g)
Control
LKO
j
Live SVF
0.062
*10^6 cells /g of fat
Control
LKO
k
CD45+
0.0068
*10^6 cells /g of fat
Control
LKO
l
Control (n=5)
LKO (n=5)
m
Neutrophils
ns
*10^6 cells /g of fat
Control
LKO
n
Eosinophils
ns
*10^6 cells /g of fat
Control
LKO
o
Monocytes
ns
*10 cells /g of fat
Control
LKO
p
Control
Acox1-LKO
0.004
0.0298
Relative mRNA expression
IL-6
IL-1b
F4/80

Supplementary Fig. 1. Characterization of glucose homeostasis and immune cells in Acox1-LKO and control mice.
a Normalized gene expression of Acox1 in livers of lean (n = 58) and obese (n = 76) patients. Data were extracted from the GEO database (GEO: GSE61260). **b** Western blot analysis of peroxisomal proteins in the liver of mice fed chow diet or a HFD (n=4). **c** Body weight of control and Acox1-LKO male mice fed normal chow diet (n=6). **d** Insulin tolerance test of control Acox1-LKO mice after HFD (n=9). **e** Pyruvate tolerance test of HFD-fed control Acox1-LKO mice (n=6). **f** Bodyweight of female mice fed a HFD (n=6 for control and 5 for Acox1-LKO). **g** Glucose tolerance test of female after HFD feeding (n=6 for control and 5 for Acox1-LKO). **h** Gating strategy for gWAT SVF flow cytometry assay. **i** Weight of gWAT fat pad for cyto-flow analysis (n=5). **j**-**k** Cell number of live SVF cells (**j**), live CD45-positive cells (**k**) per gram of gWAT from Acox1-LKO and control mice (n=5). **l** Density plots of cells in samples from control or Acox1-LKO gWAT (n=5). **m**-**o** neutrophils (**m**), eosinophils (**n**), and monocytes (**o**) per gram of gWAT from Acox1-LKO and control mice (n=5). **p** Inflammation marker genes expression in gWAT of control (n=12) and Acox1-LKO (n=13) mice after 17-weeks HFD feeding. Data in **a**-**f** and **h**-**o** are from biologically independent samples. Data are reported as the mean ± SEM. P values were determined by two-way ANOVA followed by Fisher's LSD test in **c**-**g** or two-sided unpaired Student's t test in **i**-**k**, and **m**-**p**. n.s., not significant. Source data are provided in Source Data file.

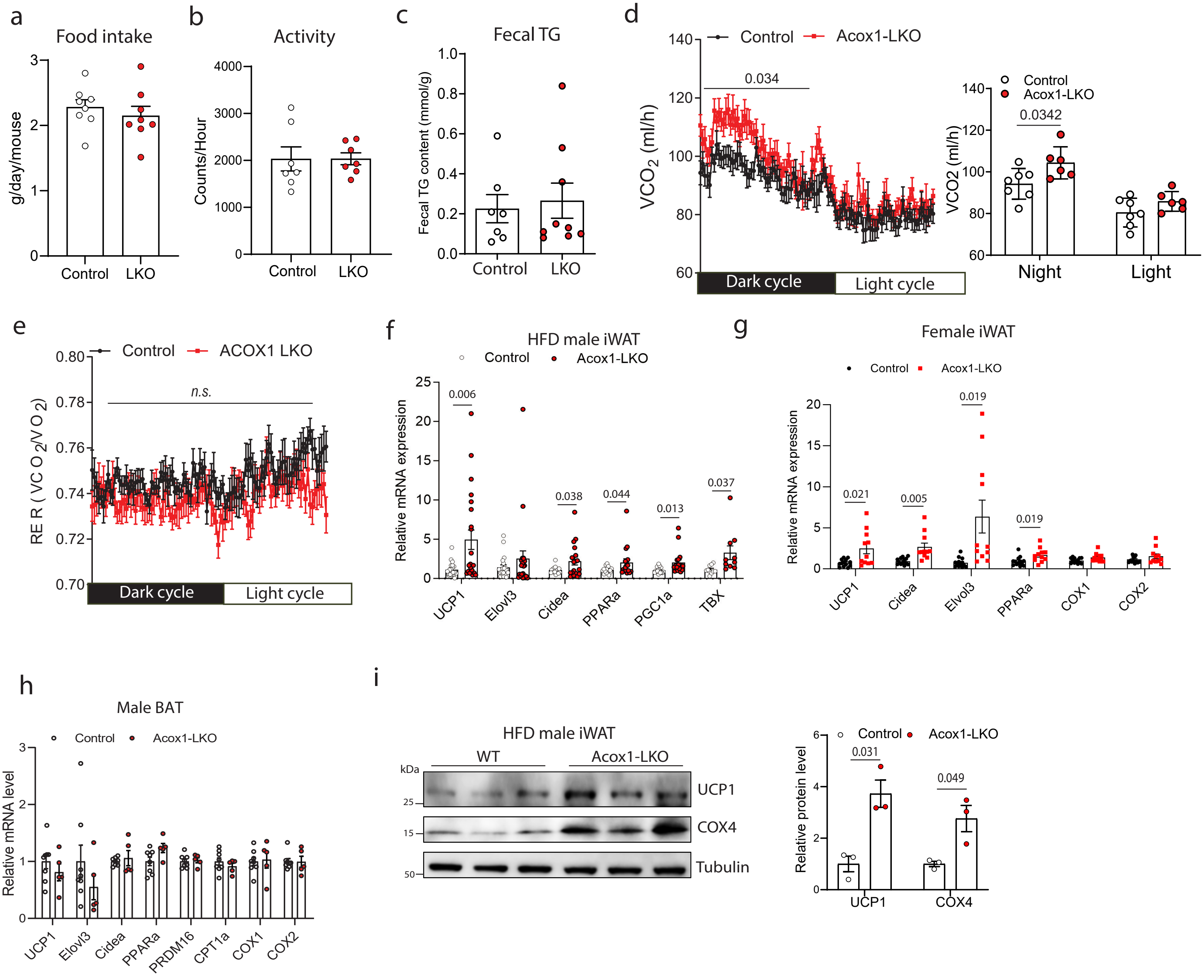

Supplementary Fig. 2. Metabolic phenotyping and adipose tissue gene expression in Acox1-LKO and control mice.
a Food intake of control and Acox1-LKO mice (n=8). **b** Locomotor activities of control and Acox1-LKO mice fed a HFD (n=7). **c** Triglyceride content of feces in HFD-fed Acox1-LKO (n=9) and control (n=7) mice. **d** VCO2 of control (n=7) and Acox1-LKO (n=6) mice. **e** Respiratory exchange ratio of control (n=7) and Acox1-LKO (n=6) mice. **f** Browning gene expression in iWAT of control and Acox1-LKO male mice fed HFD for 17 weeks [n=17 (control), 18 (Acox1-LKO) for Cidea, PPARa and PGC1a, and n= 21(control), 22 for (Acox1-LKO) for UCP1 and Elovel3, n=10 for Tbx1]. **g** Browning gene expression in iWAT of control (n=13) and Acox1-LKO (n=11) female mice under chow diet feeding. **h** Browning marker gene expression in BAT of control (n=8) and Acox1-LKO (n=5) mice fed normal chow diet. **i** UCP1 and COX4 protein in iWAT of control and Acox1-LKO mice fed HFD (n=3). Data in **a**-**i** are from biologically independent samples. Data with error bars are reported as the mean ± SEM. P values were determined by two-sided unpaired Student's t test in **a**-**c**, **d** (right panel) and **f**-**i** or two-way ANOVA with Tukey's honest difference post hoc test in **d** (left panel) and **e**. n.s., not significant. Source data are provided in Source Data file.

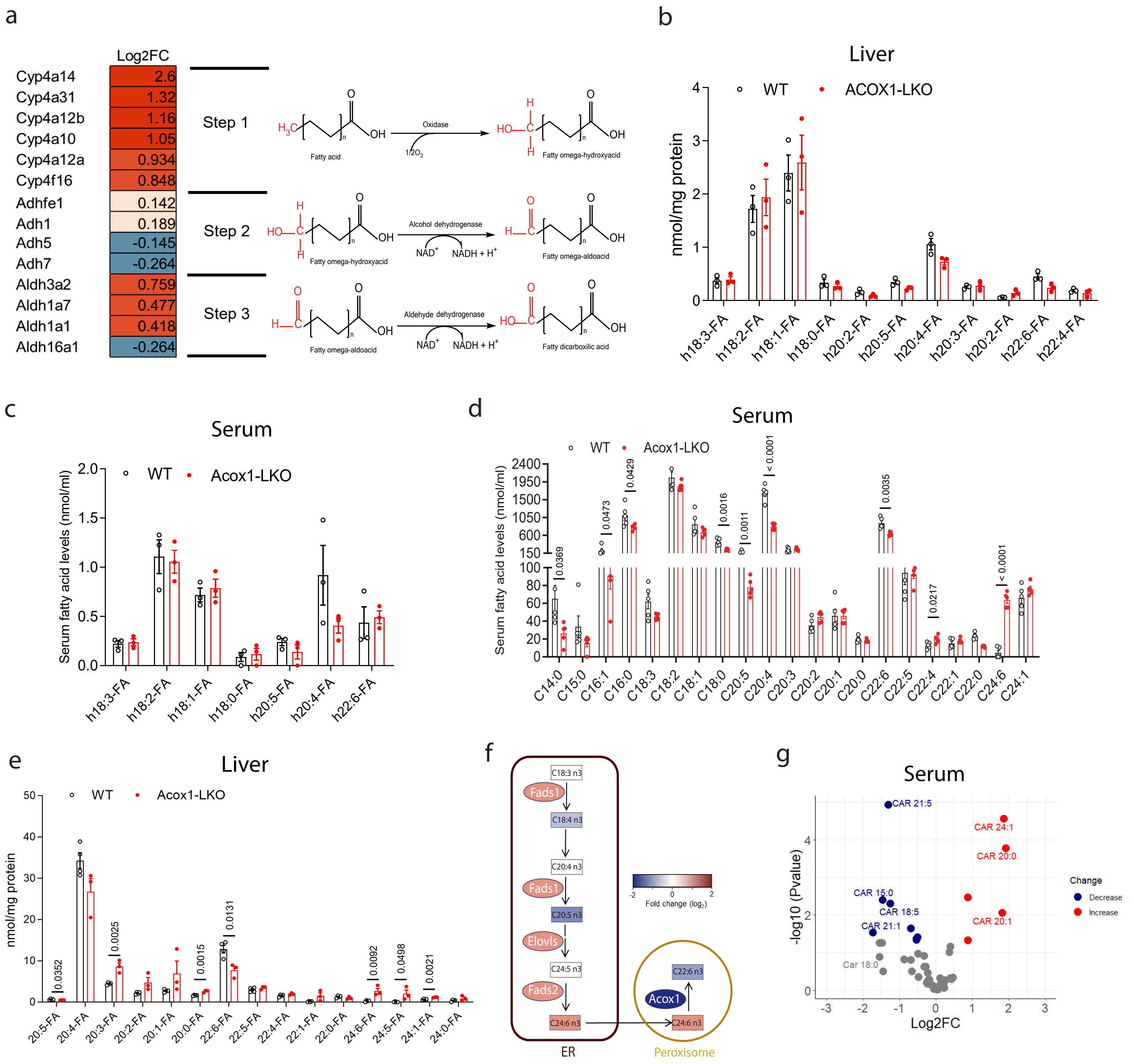

Supplementary Fig. 3. Liver and serum lipidomic analysis in Acox1-LKO and control mice.
a Genes related to fatty acid ω-oxidation in liver RNA-seq analysis. **b** Hydroxy fatty acids in the liver of control and Acox1-LKO mice (n=3). **c** Hydroxy fatty acids in serum from control and Acox1-LKO mice (n=3). **d** Total fatty acid in control and Acox1-LKO mouse serum (n=5). **e** Total fatty acid in the liver of control (n=4) and Acox1-LKO (n=3) mice. **f** Summary of major transcriptomic and lipidomic changes leading to the production of the ω-3 VLCFA C24:6. **g** Volcano plot of serum acylcarnitines. Data in **b-e** are from biologically independent samples. Data are reported as the mean ± SEM. P values were determined by two-sided unpaired Student's t test in **b-e** and **g**. n.s., not significant. Source data are provided in Source Data file.

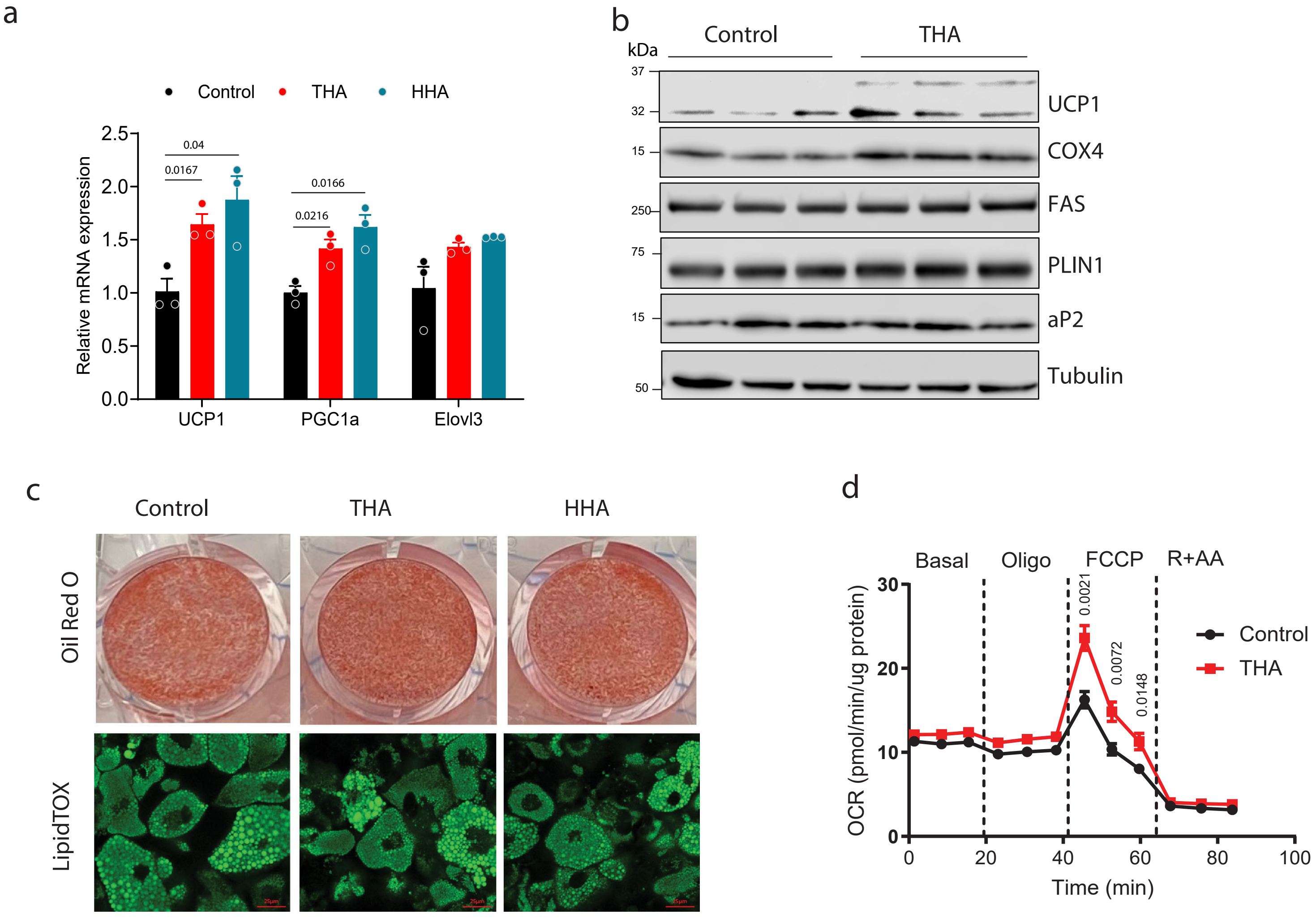

Supplementary Fig. 4. Effect of ω-3 VLCFAs on browning of adipocytes derived from human WAT.
a Browning gene expression in control, THA, or HHA-treated human adipocytes (n=3). **b** Western blot of beige and white adipocytes marker protein in control or THA-treated human adipocytes (n=3). **c** Oil Red O and LipidTox staining of control, THA or HHA-treated human adipocytes (scale bar, 25 μm). **d** OCR of control or THA-treated human adipocytes (n=6). Data in **a**-**b** and **d** are from biologically independent samples. Images in **c** are representative of two separate experiments. Data are reported as the mean ± SEM. P values were determined by two-way ANOVA followed by Fisher's LSD test in **a** and **d**. Source data are provided in Source Data file.

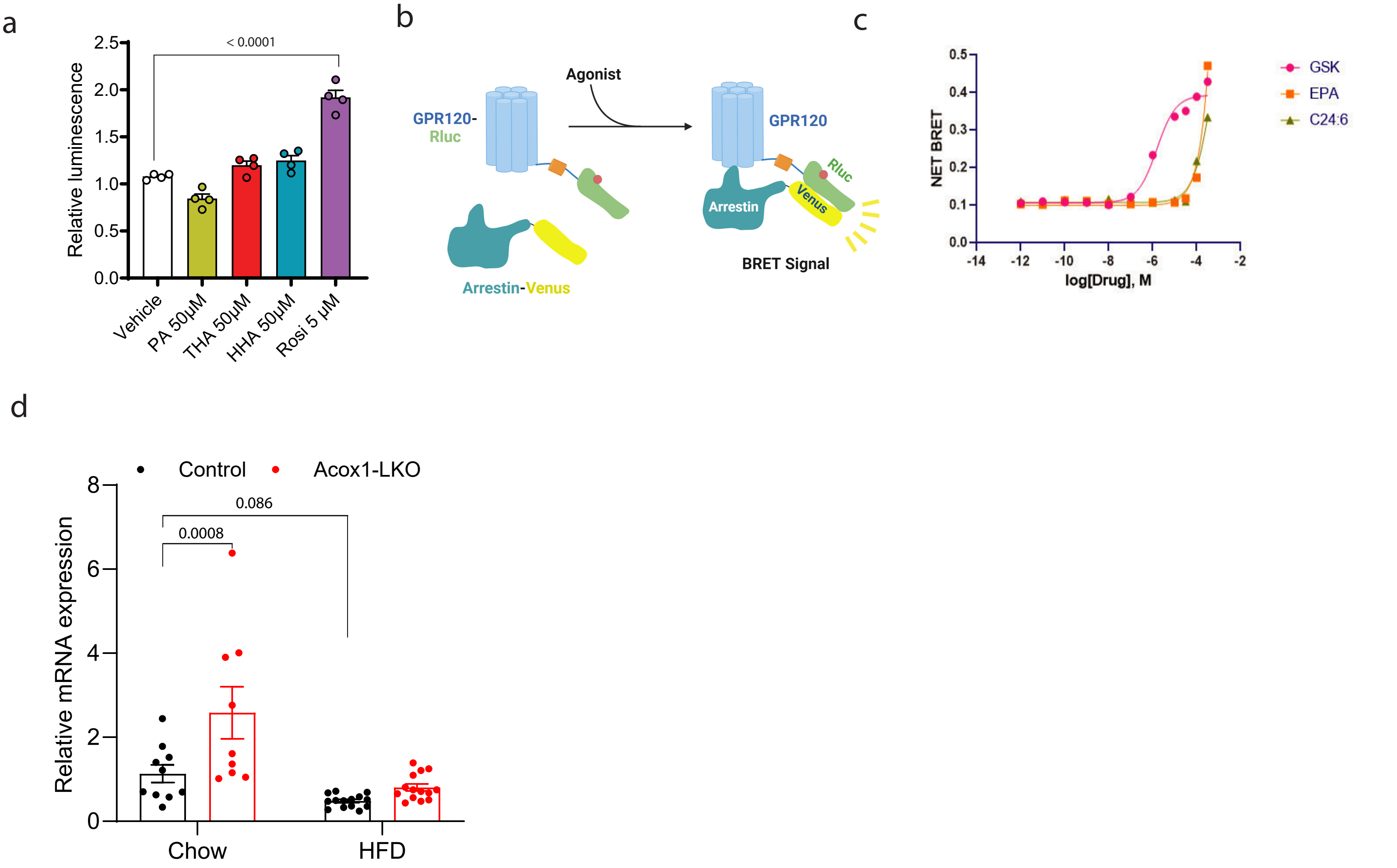

Supplementary Fig. 5. Effect of ω-3 VLCFAs on PPARγ activation and GPR120 binding.
a PPARγ-dependent luciferase reporter activity induced by vehicle, 50 µM palmitic acid, 50 µM THA, 50 µM HHA or 5 µM rosiglitazone in 293T cells expression both a PPRE-luciferase reporter construct and PPARγ (n=4). **b** Schematic of BRET assay. Created with BioRender.com. **c** BRET assay of GPR120 activation induced by GSK137647A, EPA or THA (n=2). **d** GPR120 (Ffar4) gene expression in iWAT of control and Acox1-LKO mice under chow diet or HFD feeding [n=10 (control), 9 (Acox1-LKO) for chow diet and n=14 for HFD]. Data in **a** and **c**-**d** are from biologically independent samples. Data are reported as the mean ± SEM. P values were determined by one-way ANOVA followed by Dunnett's multiple comparisons test in **a** or two-way ANOVA followed by Fisher's LSD test in **d**. Source data are provided in Source Data file.

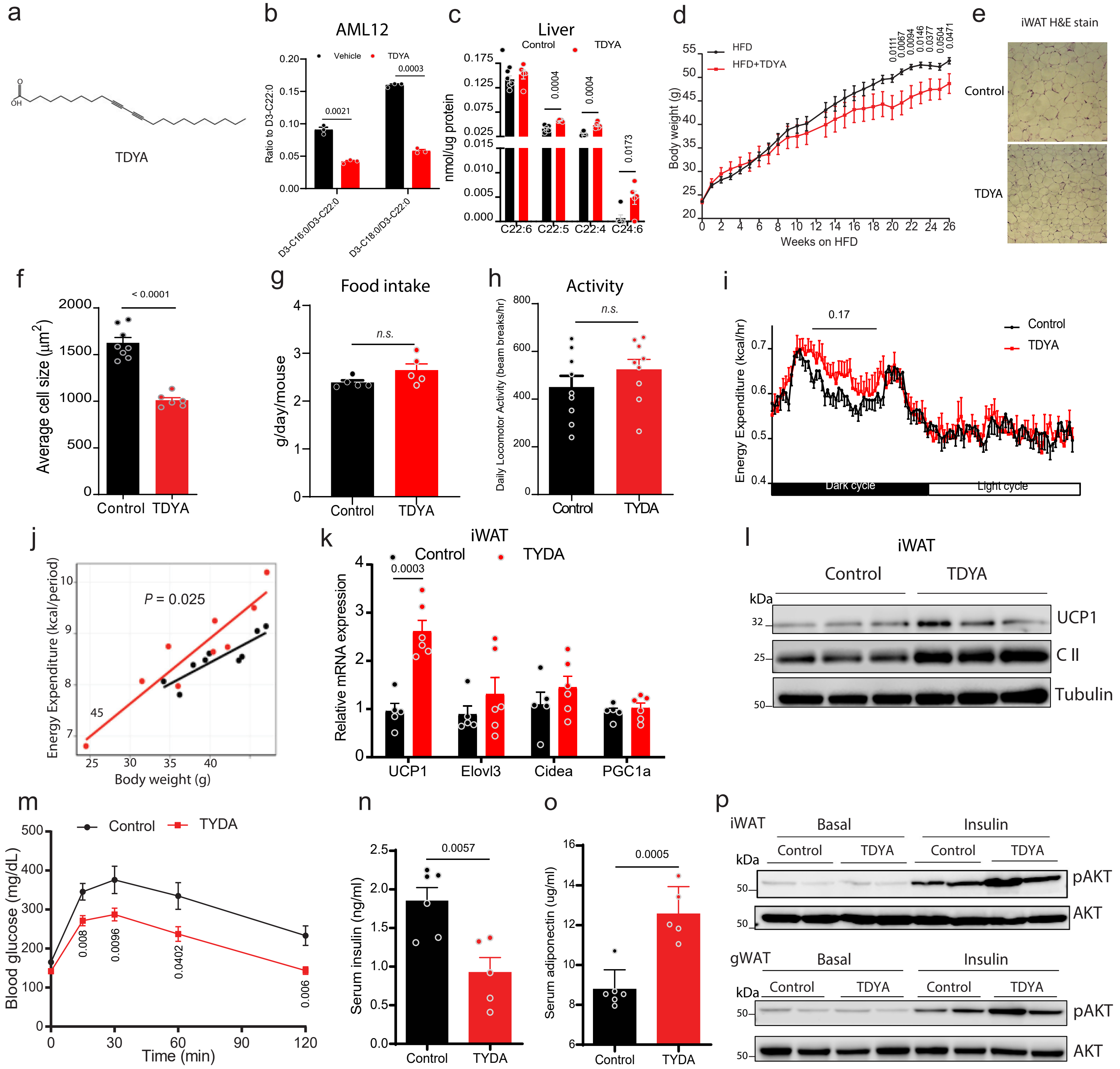

Supplementary Fig. 6. Pharmacological inhibition of ACOX1 promotes adipose tissue browning and protects mice against diet-induced obesity and insulin resistance.
a Structure of the ACOX1 inhibitor 10,12-Tricosadiynoic acid (TDYA). Created with ChemDraw. **b** Ratio of D3-C16:0 to D3-C22:0 and D3-C18:0 to D3-C22:0 in AML12 cells treated with vehicle or 30 μM TDYA (n=3). **c** Very long chain fatty acid levels in the liver of mice fed with control (n=6) or TDYA (n=5) diet. **d** Body weight of mice fed with control (n=10) or TDYA (n=9) diet. **e** H&E staining of iWAT from mice after feeding with control or TDYA diet (scale bar, 50 μm). **f** Quantification of cell size in iWAT of control (n=8) or TDYA (n=6) diet fed mice. **g** Food intake of control or TDYA diet in mice (n=5). **h** Locomotor activity of mice fed TDYA or control diet (n=9). **i** Energy expenditure of mice after feeding with control or TDYA diet (n=9). **j** Regression analysis of energy expenditure in mice fed control or TDYA diet (n=9). **k** Gene expression in iWAT of mice fed control (n=5) or TDYA (n=6) diet. **l** Levels of UCP1 and OXPHOS protein in iWAT mice fed control or TDYA diet (n=3). **m** Glucose tolerance test of control (n=10) or TDYA (n=9) fed mice. **n** Serum insulin level in control (n=6) or TDYA-(n=5) treated mice. **o** Serum adiponectin of control (n=6) and TDYA (n=5) treated mice. **p** Insulin induced Akt phosphorylation in iWAT and gWAT of control or TDYA-treated mice (n=2). Data in **b-d** and **f-p** are from biologically independent samples. Images in **e** are representative of three mice/treatment. Data are reported as the mean ± SEM. P values were determined by two-sided unpaired Student's t test in **b-c**, **f-h**, **k** and **n-o** or two-way ANOVA followed by Fisher's LSD test in **d** and **m** or two-way ANOVA with Tukey's honest difference post hoc test in **i** or ANCOVA in **j**. n.s., not significant. Source data are provided in Source Data file.

Supplementary Table 1. Oligonucleotide sequences.

Name	Forwad(5'-3')	Reverse (5'-3')
Acox1	CATGCACCATTGCCATTCGATA	CGGGAAGAGTTTATACTGCGT
Hsd17b4	AGGCTAGACTCATGGCTTCG	TGAAGTCCCCTCCTAAGTCG
Acaa1a	CAATGAACTGAAGCGTCGTG	GGTATTCAAAGACCGCAGCA
Elovl3	CTTAAGGCCCTTTTTGGAGG	CCGCGTTCTCATGTAGGTCT
Ucp1	TCAGCTGTTCAAAGCACACA	GTACCAAGCTGTGCGATGTC
Cidea	CAGTGATTTAAGAGACGCGG	TCTGCAATCCCATGAATGTC
Ppara	AGTTCGGGAACAAGACGTTG	CAGTGGGGAGAGAGGACAGA
Prdm16	CAGAGGTGTCATCCCAGGAG	ACGGATGTACTTGAGCCAGC
Pgc1a	TGTAGCGACCAATCGGAAAT	TGAGGACCGCTAGCAAGTTT
Cpt1b	GCTGCTTGCACATTTGTGTT	TGAGTGACTGGTGGGAAGAA
Cited	GCCAACCAGGAGATGAACTCTC	GATGTCGATCCAGAGGAGCTAG
Tbx1	CGAGATGATCGTCACCAAGGCA	GTCATCTACGGGCACAAAGTCC
Gpr120	CTGCCCCTCTGCATCTTGTTC	CCATGAGATTTCTCCTATGCGGTT
Gpr40	GCTTGGTCTACACTCTCCATCTG	CCAAGGCAAAGACTGGGCAGA
Gpr41	TATGTCAGCCGTGAGAGTCCG	CCAGGAGTTGAATGAAAGTCGGC
Gpr43	CCACTGTATGGAGTGATCGCTG	GGGTGAAGTTCTCGTAGCAGGT
Gpr84	AAGCCTTCCAGAAGTGCATCGC	CAGAGGAACACTGCGAAGCACA
IL-6	ACCACTTCACAAGTCGGAGGC	TTCTGCAAGTGCATCATCGTTGT
IL-1b	TGTCGGACCCATATGAGCTGAA	CACAGGTATTTTGTCGTTGCTTGG
F4/80	CTTTGGCTATGGGCTTCCAGTC	GCAAGGAGGACAGAGTTTATCGTG
Tfam	CCAAAAAGACCTCGTTCAGC	GACAGATTTTTCCAAGCCTCA
Nrf1	TAGTCCTGTCTGGGGAAACC	CTGGTACATGCTCACAGGGA
Cox1	TCCTACCACCATCATTTCTCC	CTGATGCTCCTGCATGGG
Cox2	CATCAAACCGACCAGGGTT	AATTATTGAAGCAGATCAGTTTTCG
MtNd6	CCAACATAACTCCAACATCATCA	GTATTGGGGGTGATTATAGAGGTTT
Mito-DNA	TTAAGACACCTTGCCTAGCCACAC	CGGTGGCTGGCACGAAATT
Nucler-DNA	ATGACGATATCGCTGCGCTG	TCACTTACCTGGTGCCTAGGGC
human Ucp1	CCAACTGTGCAATGAAAGTGT	CAAGTCGCAAGAAGGAAGGTA
human Pgc1a	AGTGGTGCAGTGACCAATCA	CTGCTAGCAAGTTTGCCTCA
human Elovl3	CTGTTCCAGCCCTATAACTTCG	GAATGAGGTTGCCCAATACTCC
human L-32	AAGTTCCTGGTCCACAACGTCAA	CAGCTCTTTCCACGACTTT
