# Supplementary material for: Liver ACOX1 regulates levels of circulating lipids that promote metabolic health through adipose remodeling
Source: Nat Commun. 2024 May 17;15:4214. doi: 10.1038/s41467-024-48471-2 (PMC11101658; doi:10.1038/s41467-024-48471-2)
Supplement: Supplementary file 3 — Reporting Summary [file 41467_2024_48471_MOESM3_ESM.pdf]

nature portfolio

Corresponding author(s): Irfan Lodhi

Last updated by author(s): Mar 31, 2024

Reporting Summary

Nature Portfolio wishes to improve the reproducibility of the work that we publish. This form provides structure for consistency and transparency in reporting. For further information on Nature Portfolio policies, see our Editorial Policies and the Editorial Policy Checklist.

Statistics

For all statistical analyses, confirm that the following items are present in the figure legend, table legend, main text, or Methods section.

n/a	Confirmed	
☐	☒	The exact sample size (*n*) for each experimental group/condition, given as a discrete number and unit of measurement
☐	☒	A statement on whether measurements were taken from distinct samples or whether the same sample was measured repeatedly
☐	☒	The statistical test(s) used AND whether they are one- or two-sided<br>*Only common tests should be described solely by name; describe more complex techniques in the Methods section.*
☐	☒	A description of all covariates tested
☐	☒	A description of any assumptions or corrections, such as tests of normality and adjustment for multiple comparisons
☐	☒	A full description of the statistical parameters including central tendency (e.g. means) or other basic estimates (e.g. regression coefficient) AND variation (e.g. standard deviation) or associated estimates of uncertainty (e.g. confidence intervals)
☐	☒	For null hypothesis testing, the test statistic (e.g. *F*, *t*, *r*) with confidence intervals, effect sizes, degrees of freedom and *P* value noted<br>*Give P values as exact values whenever suitable.*
☒	☐	For Bayesian analysis, information on the choice of priors and Markov chain Monte Carlo settings
☒	☐	For hierarchical and complex designs, identification of the appropriate level for tests and full reporting of outcomes
☒	☐	Estimates of effect sizes (e.g. Cohen's *d*, Pearson's *r*), indicating how they were calculated

Our web collection on statistics for biologists contains articles on many of the points above.

Software and code

Policy information about availability of computer code

Data collection	PhenoMaster data were collected by PhenoMaster TSE Systems.<br>Fat and lean mass were measured using an Echo-MRI system.<br>Fatty acid mass spec was performed by Thermo Fisher LTQ Orbitrap Velos.<br>Confocal images were acquired and processed with NIS (5.21).<br>qPCR was performed with StepOnePlus Real-Time PCR System from Applied Biosystems..<br>Flow cytometry sample acquisition and spectral unmixing were performed on Spectroflo® (3.2.1).
Data analysis	ImageJ (V1.53), Adiposoft (V1.16), GraphPad Prism (V9), R (V4.2.1), CalR (V1.3), Image Studio Lite (V 5.2), OMIQ (© 2024 Dotmatics), NovaMT LipidScreener (1.1.0) and MetaboAnalyst (4.0)

For manuscripts utilizing custom algorithms or software that are central to the research but not yet described in published literature, software must be made available to editors and reviewers. We strongly encourage code deposition in a community repository (e.g. GitHub). See the Nature Portfolio guidelines for submitting code & software for further information.

Data

Policy information about availability of data

All manuscripts must include a data availability statement. This statement should provide the following information, where applicable:

- Accession codes, unique identifiers, or web links for publicly available datasets
- A description of any restrictions on data availability
- For clinical datasets or third party data, please ensure that the statement adheres to our policy

RNA-sequencing data presented in Fig. 3 have been deposited into the NCBI GEO under the accession number GSE227896. Data of supplementary Fig. 1a were extracted from the GEO database: GSE61260. The serum lipidomic data are available in the Metabolomics Workbench database under accession number ST003142. All other data supporting this study are available within this Article and Supplemental Information. Source data are provided with this paper.

Research involving human participants, their data, or biological material

Policy information about studies with human participants or human data. See also policy information about sex, gender (identity/presentation), and sexual orientation and race, ethnicity and racism.

Reporting on sex and gender	This research did not involve human research participants.
Reporting on race, ethnicity, or other socially relevant groupings	This research did not involve human research participants.
Population characteristics	This research did not involve human research participants.
Recruitment	This research did not involve human research participants.
Ethics oversight	This research did not involve human research participants.

Note that full information on the approval of the study protocol must also be provided in the manuscript.

Field-specific reporting

Please select the one below that is the best fit for your research. If you are not sure, read the appropriate sections before making your selection.

☒ Life sciences ☐ Behavioural & social sciences ☐ Ecological, evolutionary & environmental sciences

For a reference copy of the document with all sections, see nature.com/documents/nr-reporting-summary-flat.pdf

Life sciences study design

All studies must disclose on these points even when the disclosure is negative.

Sample size	Sample size was chosen to ensure an adequate statistical power. For in vitro study, at least three biological replicates were used. The number of animal were descried in the manuscript.
Data exclusions	No data was excluded from analysis
Replication	All biological experiments were repeated at least twice and reproduced. RNA-sequencing and global lipidomic analysis experiments were performed once, but used five biologically independent samples per genotype. Selected gene expression and lipid changes were validated by qPCR and targeted lipidomics, respectively.
Randomization	For in vitro studies, conditions were randomized into control and experimental conditions as described in each assay. All animals in our experiments were randomly allocated into different groups.
Blinding	RNA-sequencing, global lipidomics and targeted lipidomics were performed by technical staff blinded to the identity of samples.

Reporting for specific materials, systems and methods

We require information from authors about some types of materials, experimental systems and methods used in many studies. Here, indicate whether each material, system or method listed is relevant to your study. If you are not sure if a list item applies to your research, read the appropriate section before selecting a response.

Materials & experimental systems

n/a	Involved in the study	
☐	☒	Antibodies
☐	☒	Eukaryotic cell lines
☒	☐	Palaeontology and archaeology
☐	☒	Animals and other organisms
☒	☐	Clinical data
☒	☐	Dual use research of concern
☒	☐	Plants

Methods

n/a	Involved in the study	
☒	☐	ChIP-seq
☐	☒	Flow cytometry
☒	☐	MRI-based neuroimaging

Antibodies

Antibodies used

Rabbit polyclonal anti-actin; Cell Signaling Technology; Cat#8457; RRID:AB_10950489; WB (1:1000)
Rabbit polyclonal anti-AKT; Cell Signaling Technology; Cat# 9272; RRID: AB_329827; WB (1:1000)
Rabbit polyclonal anti-Phospho-Akt (Ser473); Cell Signaling Technology; Cat# 927; RRID: AB_329825; WB (1:1000)
Rabbit polyclonal anti-Tubulin; Cell Signaling Technology; Cat#2146; WB (1:1000)
Mouse Monoclonal anti-COX4 (Clone: 4D11-B3-E8); Cell Signaling Technology; Cat#11967S; RRID:AB_2797784; WB (1:1000), IF (1:100)
Alexa Fluor® 647 anti-mouse F4/80 (Clone: BM8) Antibody; Biolegend; Cat# 123121; RRID:AB_893492; IF (1:100)
Rabbit polyclonal anti-Acox1; Proteintech; Cat# 10957-1-AP; RRID: AB_2221670; WB (1:1000)
Mouse monoclonal anti-c-Myc (Clone: 9E10); Santa cruz; Cat#SC-40; RRID:AB_627268; IF (1:100)
Rabbit polyclonal anti-UCP1; Abcam; Cat#ab23841; RRID:AB_2213764; WB(1:1000), IF (1:100)
Total OXPHOS Rodent WB Antibody Cocktail; Abcam; Cat#ab110413; RRID:AB_2629281; WB (1:400)
Rabbit Monoclonal anti-Tom20 (Clone: D8T4N); Cell Signaling Technology; Cat#42406; RRID:AB_2687663; WB (1:1000)
Rabbit polyclonal anti-PEX16; proteintech; Cat#14816-1-AP; RRID:AB_2162250; WB (1:1000)
Rabbit polyclonal anti-PEX5; proteintech; Cat#12545-1-AP; RRID:AB_2268102; WB (1:1000)
Mouse monoclonal Anti-PMP70 (Clone: 70-18) antibody; sigma; Cat#SAB4200181; RRID:AB_10639362; WB (1:1000)
Rabbit polyclonal anti-FASN; Proteintech; Cat# 10624-2-AP; RRID:AB_2100801; WB (1:5000)
Rabbit monoclonal anti-perilipin-1 (Clone: D1D8); Cell Signaling Technology; Cat#9349; WB (1:1000)
Rabbit polyclonal anti-FABP4; Cell Signaling Technology; Cat#2120; WB (1:1000)
Peroxidase IgG Fraction Monoclonal Mouse Anti-Rabbit IgG, light chain specific; Jackson Immunoresearch; Cat# 211-032-171; RRID: AB_2339149; WB (1:2000)
Peroxidase AffiniPure Goat Anti-Mouse IgG, light chain specific; Jackson Immunoresearch; Cat# 115-035-174; RRID: AB_2338512; WB (1:2000)
Goat anti-Rabbit IgG (H+L) Cross-Adsorbed Secondary Antibody, Alexa Fluor 594; Thermo Fisher; Cat# A-11012; RRID:AB_141359; IF (1:500)
Goat anti-mouse IgG (H+L) Cross-Adsorbed Secondary Antibody, Alexa Fluor 488; Thermo Fisher; Cat# A-11001; RRID:AB_2534069; IF (1:500)
Anti-Mouse CD45; BD Biosciences; Cat#566095; RRID:AB_2739499; FC (1μg/ml)
Brilliant Violet 650™ anti-mouse CD3 [Clone: 17A2]; Biolegend; Cat#100229; RRID:AB_11204249; FC (1μg/ml)
PE/Dazzle™ 594 anti-mouse CD4 [Clone: RM4-5]; Biolegend; Cat#100566; RRID:AB_2563685; FC (1μg/ml)
PE anti-mouse CD8a [Clone: 53-6.7]; Biolegend; Cat#100708; RRID:AB_312747; FC (0.4μg/ml)
APC anti-mouse TCR γ/δ[Clone: GL3]; Biolegend; Cat#118116; RRID:AB_1731813; FC (1μg/ml)
Brilliant Violet 711™ anti-mouse CD19 [Clone: 6D5]; Biolegend ; Cat#115555; RRID:AB_2565970; FC (1μg/ml)
PE/Cyanine7 anti-mouse F4/80 [Clone: BM8]; Biolegend; Cat#123114; RRID:AB_893478; FC (1μg/ml)
Brilliant Violet 605™ anti-mouse/human CD11b [Clone: M1/70]; Biolegend; Cat#101257; RRID:AB_2565431; FC (1μg/ml)
Anti-CD9 BV786 KMC8; BD Biosciences; Cat#740886; RRID:AB_2740535; FC (1μg/ml)
Brilliant Violet 570™ anti-mouse Ly-6C [Clone: HK1.4]; Biolegend; Cat#128029; RRID:AB_10896061; FC (0.5μg/ml)
PE/Cyanine5 anti-mouse CD11c [Clone: N418]; Biolegend; Cat#117316; RRID:AB_493566; FC (1μg/ml)
Alexa Fluor® 700 anti-mouse CD206 (MMR) [Clone: C068C2]; Biolegend; Cat#141734; RRID:AB_2629637; FC (5μg/ml)
Spark Blue™ 550 anti-mouse I-A/I-E [Clone: M5/114.15.2]; Biolegend; Cat#107662; RRID:AB_2860616; FC (2.5μg/ml)
Ms NK-1.1 BV750 PK136; BD Biosciences; Cat#746876; RRID:AB_2871676; FC (2μg/ml)
FITC anti-mouse Ly-6G [Clone: 1A8]; Biolegend; Cat#127606; RRID:AB_1236494; FC (1μg/ml)
Ms FceR1a BV510 MAR-1; BD Biosciences; Cat#751757; RRID:AB_2875734; FC (1μg/ml)
Brilliant Violet 421™ anti-mouse/rat XCR1 [Clone: ZET]; Biolegend; Cat#148216; RRID:AB_2565230; FC (1μg/ml)
Ms Siglec-F APC-Cy7 E50-2440; BD Biosciences; Cat#565527; RRID:AB_2732831; FC (1μg/ml)
PerCP/Cyanine5.5 anti-mouse/human CD44 [Clone: IM7]; Biolegend; Cat#103032; RRID:AB_2076204; FC (0.67μg/ml)

Validation

Anti-Actin (https://www.cellsignal.com/products/primary-antibodies/b-actin-d6a8-rabbit-mab/8457)
Reactivity: Human, Mouse, Rat, Monkey, D. melanogaster, Zebrafish
Applications: WB, IF, FC
Anti-AKT (https://www.cellsignal.com/products/primary-antibodies/akt-antibody/9272)
Reactivity: Human, Mouse, Rat, Hamster, Monkey, Chicken, D. melanogaster, Bovine, Dog, Pig, Guinea Pig
Applications: WB, ,IP, IF, FC
Anti-phospho-Akt (Ser473) (https://www.cellsignal.com/products/primary-antibodies/phospho-akt-ser473-antibody/9271)

Reactivity: Human, Mouse, Rat, Hamster, Monkey, D. melanogaster, Bovine, Dog
Applications: WB, ,IP, IF, FC
Anti-Tubulin(https://www.cellsignal.com/products/primary-antibodies/b-tubulin-antibody/2146)
Species Reactivity: Human, Mouse, Rat, Monkey, Zebrafish, Bovine
Applications: WB, ,IP, IF, FC
Anti-COX4 (https://www.cellsignal.com/products/primary-antibodies/cox-iv-4d11-b3-e8-mouse-mab/11967)
Species Reactivity:Human, Mouse, Rat, Monkey
Applications: WB, ,IP, IHC, IF
Anti-F4/80(https://www.biolegend.com/en-gb/products/alexa-fluor-647-anti-mouse-f4-80-antibody-4074?GroupID=BLG5319)
Verified Reactivity:Mouse
Applications: FC, IHC-F, 3D-IHC
Anti-Acox1(https://www.ptglab.com/products/AOX-Antibody-10957-1-AP.htm)
Reactivity: Human, Mouse, Rat
Applications: WB, IHC, IF, ELISA
Anti-Myc(https://www.scbt.com/p/c-myc-antibody-9e10)
Reactivity: mouse, rat, human, monkey
Applications: WB, IP, IF, IHC(P), FCM and ELISA
Anti-UCP1(https://www.abcam.com/products/primary-antibodies/ucp1-antibody-ab23841.html)
Reactivity: Mouse, Rat; Predicted to work with: Hamster, Dog, Human, Chimpanzee
Applications: WB
Total OXPHOS Rodent WB Antibody Cocktail(https://www.abcam.com/products/panels/total-oxphos-rodent-wb-antibody-cocktail-ab110413.html)
Reactivity: Mouse, Rat, Cow, Human, Cynomolgus monkey
Applications: WB
Anti-Tom20(https://www.cellsignal.com/products/primary-antibodies/tom20-d8t4n-rabbit-mab/42406)
Species Reactivity: Human, Mouse, Rat, Monkey
Applications: WB, IP, IF, IHC(P),
Anti-PEX16(https://www.ptglab.com/products/PEX16-Antibody-14816-1-AP.htm)
Reactivity: Human, Mouse, Rat
Applications: WB, IF
Anti-PEX5(https://www.ptglab.com/products/PEX5-Antibody-12545-1-AP.htm)
Reactivity: Human, Mouse, Rat
Applications: WB, IP, IHC, IF, FC, ELISA
Anti-PMP70(https://www.sigmaaldrich.com/US/en/product/sigma/sab4200181)
Reactivity: Human, Mouse, Rat
Applications: WB, IF
Anti-FASN(https://www.ptglab.com/products/FASN-Antibody-10624-2-AP.htm)
Reactivity: Human, Mouse, Rat
Applications: WB, IP, IHC, IF, FC, CoIP, ELISA
Anti-perilipin-1(https://www.cellsignal.com/products/primary-antibodies/perilipin-1-d1d8-xp-rabbit-mab/9349)
Species Reactivity: Human, Mouse
Applications: WB, IP, IHC, IF,
Anti-FABP4(https://www.cellsignal.com/products/primary-antibodies/fabp4-antibody/2120)
Species Reactivity: Human, Mouse
Application: WB
Anti-CD45(https://www.bdbiosciences.com/en-us/products/reagents/flow-cytometry-reagents/research-reagents/single-color-antibodies-ruo/bv480-rat-anti-mouse-cd45.566095)
Reactivity: Mouse
Application: FC
Anti-CD3(https://www.biolegend.com/fr-ch/products/brilliant-violet-650-anti-mouse-cd3-antibody-7843)
Reactivity: Mouse
Application: FC
Anti-CD4(https://www.biolegend.com/fr-lu/products/pe-dazzle-594-anti-mouse-cd4-antibody-9845?GroupID=BLG4211)
Reactivity: Mouse
Application: FC
Anti-CD8a(https://www.biolegend.com/fr-fr/products/pe-anti-mouse-cd8a-antibody-155)
Reactivity: Mouse
Application: FC
Anti-TCR γ/δ(https://www.biolegend.com/ja-jp/search-results/apc-anti-mouse-tcr-gamma-delta-antibody-6061?GroupID=BLG3687)
Reactivity: Mouse
Application: FC
Anti-Cd19(https://www.biolegend.com/de-at/products/brilliant-violet-711-anti-mouse-cd19-antibody-12075)
Reactivity: Mouse
Application: FC
Anti-F4/80(https://www.biolegend.com/fr-fr/productstab/pe-cyanine7-anti-mouse-f4-80-antibody-4070?GroupID=BLG5319)
Reactivity: Mouse
Application: FC
Anti-CD11b(https://www.biolegend.com/de-at/products/brilliant-violet-605-anti-mouse-human-cd11b-antibody-7637)
Reactivity: Chimpanzee, Baboon, Rabbit

Application: FC
Anti-CD9(https://www.bdbiosciences.com/en-ch/products/reagents/flow-cytometry-reagents/research-reagents/single-color-antibodies-ruo/bv786-rat-anti-mouse-cd9.740886)
Reactivity: Mouse
Application: FC
Anti- Ly-6C(https://www.biolegend.com/nl-be/products/brilliant-violet-570-anti-mouse-ly-6c-antibody-7392?GroupID=BLG5853)
Reactivity: Mouse
Application: FC
Anti-CD11c(https://www.biolegend.com/en-gb/products/pe-cyanine5-anti-mouse-cd11c-antibody-3085?GroupID=BLG11937)
Reactivity: Mouse
Application: FC
Anti-CD206(https://www.biolegend.com/nl-be/products/alexa-fluor-700-anti-mouse-cd206-mmr-antibody-13456)
Reactivity: Mouse
Application: ICFC, FC
Anti-I-A/I-E(https://www.biolegend.com/en-gb/products/spark-blue-550-anti-mouse-i-a-i-e-antibody-19227?GroupID=BLG4736)
Reactivity: Mouse
Application: FC
Anti-NK-1.1(https://www.bdbiosciences.com/en-us/products/reagents/flow-cytometry-reagents/research-reagents/single-color-antibodies-ruo/bv750-mouse-anti-mouse-nk-1-1.746876)
Reactivity: Mouse
Application: FC
Anti-Ly-6G(https://www.biolegend.com/fr-lu/explore-new-products/fitc-anti-mouse-ly-6g-antibody-4775)
Reactivity: Mouse
Application: FC
Anti-FceR1a(https://www.bdbiosciences.com/en-us/products/reagents/flow-cytometry-reagents/research-reagents/single-color-antibodies-ruo/BV510-Hamster-Anti-Mouse-FceR1a.751757)
Reactivity: Mouse
Application: FC
Anti-XCR1(https://biolegend.com/nl-be/products/brilliant-violet-421-anti-mouse-rat-xcr1-antibody-10750?GroupID=GROUP20)
Reactivity: Mouse, Rat
Application: FC
Anti-Siglec-F(https://www.bdbiosciences.com/en-us/products/reagents/flow-cytometry-reagents/research-reagents/single-color-antibodies-ruo/apc-cy-7-rat-anti-mouse-siglec-f.565527)
Reactivity: Mouse, Rat
Application: FC
Anti-CD44(https://www.biolegend.com/nl-be/products/percp-cyanine5-5-anti-mouse-human-cd44-antibody-5605)
Reactivity: Mouse, Human
Application: FC

Eukaryotic cell lines

Policy information about cell lines and Sex and Gender in Research

Cell line source(s)	Immortalized mouse iWAT preadipocytes were isolated in the Lodhi lab and have been previously described. Immortalized human white preadipocytes were generated in the Tseng lab and have been previously described. HEK293T and AML12 cells were from ATCC.
Authentication	We did not make efforts to authenticate cell lines.
Mycoplasma contamination	All cell lines were tested negative for mycoplasma.
Commonly misidentified lines (See ICLAC register)	No commonly misidentified cell were studied.

Animals and other research organisms

Policy information about studies involving animals; ARRIVE guidelines recommended for reporting animal research, and Sex and Gender in Research

Laboratory animals	WT C57BL/6J, Acox1-LKO and albumin-cre mice were used in experiments starting at 8 to 10 weeks of age. The Acox1-LKO and albumin-cre mice were generated on the C57BL/6J genetic background. All mice were maintained under constant temperature (23-25°C), circulating air and humidity (45-65%) with 12h:12h light/dark cycle. Mice had free access to food and water.
Wild animals	No wild animals were used in this study.
Reporting on sex	Both male and female animals were studied for metabolic phenotyping studies, as indicated in figure legends.
Field-collected samples	No field collected samples were used in the study.
Ethics oversight	All animal protocols were approved by the Washington University Institutional Animal Care and Use Committee.

Note that full information on the approval of the study protocol must also be provided in the manuscript.

Plants

Seed stocks	*Report on the source of all seed stocks or other plant material used. If applicable, state the seed stock centre and catalogue number. If plant specimens were collected from the field, describe the collection location, date and sampling procedures.*
Novel plant genotypes	*Describe the methods by which all novel plant genotypes were produced. This includes those generated by transgenic approaches, gene editing, chemical/radiation-based mutagenesis and hybridization. For transgenic lines, describe the transformation method, the number of independent lines analyzed and the generation upon which experiments were performed. For gene-edited lines, describe the editor used, the endogenous sequence targeted for editing, the targeting guide RNA sequence (if applicable) and how the editor was applied.*
Authentication	*Describe any authentication procedures for each seed stock used or novel genotype generated. Describe any experiments used to assess the effect of a mutation and, where applicable, how potential secondary effects (e.g. second site T-DNA insertions, mosiacism, off-target gene editing) were examined.*

Flow Cytometry

Plots

Confirm that:

- [x] The axis labels state the marker and fluorochrome used (e.g. CD4-FITC).
- [x] The axis scales are clearly visible. Include numbers along axes only for bottom left plot of group (a 'group' is an analysis of identical markers).
- [x] All plots are contour plots with outliers or pseudocolor plots.
- [x] A numerical value for number of cells or percentage (with statistics) is provided.

Methodology

Sample preparation	Cells were collected by digesting fresh gonadal adipose tissue with DMEM containing 0.5% BSA and 1 mg/mL collagenase D for 30 min at 37°C. Upon digestion, cells were sieved through 100 µm cell strainers and spun down to collect the pellet containing stromal vascular fraction (SVF). Red blood cells were removed by incubating in lysis buffer (#A1049201, Gibco) for 3 min. Then cells were incubated with Live-or-Dye 665/685 viability dye (Biotium). After washing and blocking with TruStain FcX PLUS (2.5 µg/mL; #156604 Biolegend), cells were then stained with the murine cocktail of antibodies CD45 BV480, CD3 BV650, CD4 PE/Dazzle 594, CD8a PE, TCR g/d APC, F4/80 PE/Cyanine7, CD11b BV605, CD9 BV786, Ly-6C BV570, CD11c PE/Cyanine5, CD206 Alexa Fluor 700, I-A/I-E Spark Blue 550, NK-1.1 BV750, Ly-6G FITC, FceR1a BV510, XCR1 BV421, siglec-F APC-Cy7, CD44 PerCP/Cyanine5.5 for 30 min at 4°C. Then cells were fixed using eBioscience™ Foxp3 / Transcription Factor Staining Buffer Set. Finally cells were washed and resuspended in ice-cold PBS for acquisition.
Instrument	Cytek Northern Lights 3-laser spectral cytometer
Software	SpectroFlo for sample aquisition and spectral unmixing, and OMIQ platform for FCS file analysis
Cell population abundance	After SVF collection and RBC lysis, we counted total SVF using BioRad TC20 automated cell counter. We used Live-or-Dye 665/685 viability dye (Biotium) and CD45 BV480 to measure the percentage of CD45 cells among all live SVF cells. We then multiplied the % of live CD45 by the SVF count and divided this value by the fat pad weight to measure the abundance of CD45 per gram of fat.
Gating strategy	Doublets and debris were excluded from the analysis using FSC/SSC plots. Dead cells were excluded using Live dead positive control. Live CD45+ cells were downsampled to 48774 cells per sample. Uniform manifold approximation and projection (UMAP) was used to visualize the data. FlowSOM algorithm was used to generate filters, which were then manually inspected and adjusted in some cases.

- [x] Tick this box to confirm that a figure exemplifying the gating strategy is provided in the Supplementary Information.
